# Review of "Understanding the Human Machine, A Primer for Bioengineering" by Max E. Valentinuzzi

**DOI:** 10.1186/1475-925X-4-8

**Published:** 2005-02-10

**Authors:** Tzung K Hsiai

**Affiliations:** 1 Department of Biomedical Engineering and Division of Cardiovascular Engineering, University of Southern California, 1042 Downey Way, Denney Research Center (DRB) 140, Los Angeles, CA 90089-1111, USA

The book "Understanding the Human Machine, A Primer for Bioengineering" embraces various aspects of biomedical engineering as an essential resource book for physical scientists, engineers and biomedical students. The book interfaces physiologic systems with engineering principles to capture the important concepts from a plethora of facts in field of biomedical sciences. Humors, exercise, history of biomedical discovery and elements of humility continuously emerge throughout the book; thereby, enhancing the self-taught approach for the new comers.

The Introduction reflects the author's philosophy and advice for the readers from his accumulated wisdom and experience in teaching bioengineering. In Chapter 2, the author used the system approach to intertwine cardiovascular, renal, respiratory, gastrointestinal, endocrine, nervous and muscular systems. The organization is timely and effective in the era of systems biology and the emerging research towards predictive and preventive medicine.

The system approach in Chapter 2 paves the way for detecting signal output from the physiologic systems. Biosensors are illustrated to measure electric signals from the visual, auditory and olfactory systems as well as the heart, brain, and muscle. The integration of electric circuits using the classic examples such as the Wheatstone bridge and operational amplifiers provides the frame work for signal acquisition and processing. Finally, the author completed the book with a touch of bioinformatics in the post genomic era.

In general, the book is comprehensive and succinct, readable and interesting. The presented material spans from the fundamental principles to the applications of bioengineering.

Certain areas will improve the quality of this book for the readership of undergraduates and new comers to bioengineering. Some figures are difficult to understand as a result of small captions (eg. figs 2.11 and 3.19). Others are old and lack clear labeling (eg. Figs. 2.52 and 3.15). Tissue engineering deserves a paragraph. Analogous to nanotechnology, a link to a tissue engineering website would be helpful for the readers. A paragraph on molecular engineering involving proteins, DNA and RNA molecules would enhance the balance between classic bioengineering and the emerging fields. The readers would be intrigued if the book ends with a paragraph introducing Bio-MEMS (Micro Electrical Mechanical Systems), bionanotechnology, molecular imaging, and surface chemistry.

Overall, "Understanding the Human Machine, A Primer for Bioengineering" is a very useful book that highlights the fusion between organ systems and engineering principles. I would recommend this book as an introduction to bioengineering and a reference for students from physical science, engineering and life science.

